# Impact of Stroke Code Activation on Functional Outcomes and the Role of Nursing in Neurorehabilitation: A Systematic Review

**DOI:** 10.3390/neurolint17110175

**Published:** 2025-10-29

**Authors:** Álvaro Astasio-Picado, Jesus Jurado-Palomo, Clara Fátima Rodriguez-Urbaneja

**Affiliations:** 1 Physiotherapy, Nursing and Physiology Department, Faculty of Health Sciences, University of Castilla-La Mancha, 45600 Toledo, Spain; jesus.juradopalomo@uclm.es (J.J.-P.); clarafatima.urbaneja@gmail.com (C.F.R.-U.); 2 Department of Nursing, University Center of Plasencia, University of Extremadura, 10600 Cáceres, Spain

**Keywords:** Stroke Code, ischemic stroke, time prognosis, neurorehabilitation, nursing

## Abstract

**Introduction:** Stroke is one of the leading causes of death and disability worldwide. In this context, early activation of the Stroke Code and a structured neurorehabilitation approach are key determinants of patients’ functional outcomes. **Objectives:** We aimed to evaluate the impact of Stroke Code activation on the functional prognosis of patients who have suffered an ischemic stroke, analyzing the time-dependent relationship and the effectiveness of reperfusion therapies. Additionally, we sought to examine the role of nursing in inpatient neurorehabilitation. **Methods:** A systematic review was conducted following the PRISMA 2020 guidelines. Scientific studies published between 2020 and 2025 were reviewed across five databases: PubMed; Cochrane Library; Dialnet; Web of Science; and Scopus. Eligibility criteria were applied, and validated tools were used to assess methodological quality and risk of bias. **Results:** Thirteen studies were included, involving a total sample of 80,555 patients. Age; lesion volume; and time to treatment were found to be key prognostic factors. Early implementation of reperfusion therapies (thrombolysis and/or thrombectomy), combined with nursing-led neurorehabilitation interventions, significantly improved neurological status, functional independence, and quality of life. **Conclusions:** Stroke Code activation has a significant positive influence on functional prognosis. Reducing treatment delays and optimizing reperfusion therapies are critical. Furthermore, the role of nursing in hospital-based neurorehabilitation is essential to support patient recovery and functionality.

## 1. Analysis and Justification

Stroke is currently one of the most prevalent and impactful neurological conditions worldwide, both in terms of mortality and long-term disability. Its clinical relevance and socioeconomic burden make it a priority area for public health systems and medical research. The acute nature of strokes, particularly ischemic strokes, demands immediate intervention, as the progression of irreversible brain damage begins within minutes of vascular occlusion. Scientific evidence has been used to estimate that for every minute that cerebral perfusion is compromised, approximately 1.9 million neurons are lost, 14 billion synapses are disrupted, and 12 km of myelinated fibers are destroyed—reinforcing the commonly used phrase: “time is brain” [1].

Globally, the data is striking. In 2020, stroke was the second leading cause of death, accounting for 6.6 million deaths, and the third leading cause of disability, responsible for 143 million disability-adjusted life years (DALYs) [2]. In Europe, projections indicate a continued increase in stroke incidence due to aging populations, with Spain reporting around 120,000 new cases each year and approximately 25,000 related deaths. In 2019, stroke was responsible for 461,645 DALYs in Spain, representing 3.9% of all DALYs from all causes. Forecasts predict a 35% increase in this burden by 2035, which underlines the urgency of improving stroke care systems and preventive strategies [3].

The economic impact is equally significant. The total estimated cost of incident strokes in Spain amounts to € 1.989 billion annually. On an individual level, the average cost per patient during the first year after the event is estimated at € 27,711, which includes hospitalization, pharmacological treatment, rehabilitation, and loss of productivity [3]. These data reinforce the need for efficient and integrated systems of care that minimize neurological damage, reduce the duration of hospital stays, and promote functional recovery.

In this context, the implementation of the Stroke Code (SC) represents one of the most impactful public health measures in recent years. The SC refers to a structured and multidisciplinary set of actions designed to ensure early recognition, rapid activation of emergency services, expedited diagnosis, and timely administration of reperfusion therapies—mainly intravenous thrombolysis and mechanical thrombectomy—especially for patients who have suffered an ischemic stroke [3,4,5,6,7,8,9,10,11]. Numerous studies have demonstrated that the probability of a favorable outcome increases significantly when therapeutic windows are respected and when interventions are carried out in stroke-ready centers with specialized personnel [10,12].

However, it is not only the acute-phase response that determines the prognosis. Stroke is not a one-time event but rather a life-altering condition whose recovery trajectory can be unpredictable and non-linear. Approximately 50% of stroke survivors are left with some degree of disability, and more than one-third experience severe limitations in their daily activities. The most common sequelae include motor deficits (50–85%), cognitive impairments (50%), communication disorders (33%), and neuropsychiatric complications such as depression and anxiety [3,13,14]. These functional and psychological consequences highlight the need for continuity of care beyond the emergency phase.

Early and structured neurorehabilitation has proven to be a key determinant in restoring independence and reducing disability after a stroke. The initiation of therapeutic interventions within the first 24–72 h—preferably in dedicated Stroke Units (SUs)—is associated with better neurological recovery, reduced complications, and improved reintegration into daily life [11,13,15]. Within these units, nursing professionals play a vital role, not only in hemodynamic and neurological monitoring but also in implementing rehabilitative strategies tailored to each patient’s needs. Their involvement in mobilization protocols, dysphagia management, sensory stimulation, patient education, and emotional support has shown positive effects on recovery trajectories [15,16].

Despite the growing body of evidence supporting the SC model and the effectiveness of nursing interventions, comprehensive reviews analyzing the combined influence of both factors on functional prognosis are still limited. Therefore, the present systematic review aims to explore the impact of the Stroke Code on the functional outcomes of patients who have suffered an ischemic stroke, with particular attention to the time-dependent nature of recanalization therapies. Furthermore, it seeks to assess the role of hospital-based nursing care in neurorehabilitation and how such interventions contribute to neurological recovery, reductions in disability, and improvement of the patient’s quality of life.

By synthesizing the scientific literature published between 2020 and 2025 and adhering to PRISMA 2020 guidelines, this review aspires to offer an updated and evidence-based perspective that can inform future clinical practice, optimize decision-making, and reinforce the importance of a comprehensive, interdisciplinary approach to stroke care.

## 2. Methodology

### 2.1. Study Design

This study corresponds to a systematic review of the scientific literature, conducted according to the PRISMA 2020 (Preferred Reporting Items for Systematic Reviews and Meta-Analyses) guidelines. The protocol was previously registered in the PROSPERO international prospective register of systematic reviews (ID: CRD420251035656) [17].

The review included recent and relevant studies published between 2020 and 2025, addressing the research objectives from diverse methodological perspectives.

The data extraction process was conducted by two independent reviewers. The first reviewer performed the initial extraction, and the second verified the accuracy and consistency of the data, making corrections when discrepancies arose.

### 2.2. Research Questions

Two research questions were developed based on the PICO model:

1. In patients with ischemic stroke, how do interventions implemented through the Stroke Code (SC) affect functional outcomes?
2. How do nursing interventions influence the effectiveness of the inpatient rehabilitation process and the patient’s quality of life?

PICO elements:

- P (Population): Adult patients with ischemic stroke.
- I (Intervention): Interventions under the Stroke Code and during inpatient rehabilitation.
- C (Comparison): Comparison of functional outcomes depending on the type of care provided.
- (Outcome): Functional prognosis, measured by post-stroke sequelae and degree of disability.

### 2.3. Information Sources

The following electronic databases were systematically searched for relevant studies, with the last search dates as follows:

- Dialnet: January 12, 2025
- Scopus: January 17, 2025
- Cochrane Library: January 17, 2025
- Web of Science (WoS): February 5, 2025
- PubMed: February 10, 2025

### 2.4. Eligibility Criteria

Inclusion Criteria:

- Publications from the last 5 years (2020–2025)
- Languages: Spanish and English
- Thematic relevance: Studies addressing the Stroke Code, functional prognosis, treatment, rehabilitation, and nursing
- Full-text availability
- Study design: Systematic reviews, meta-analyses, randomized controlled trials (RCTs), and cohort studies

Exclusion Criteria:

- Publications prior to 2020
- Languages other than Spanish or English
- Studies without full-text access
- Duplicate publications or studies with nonspecific outcomes
- Studies with low methodological quality or high risk of bias (assessed through validated tools)

### 2.5. Types of Studies Included

- To ensure a comprehensive and rigorous approach to the research questions, the review included studies of the following designs:
- Systematic reviews and meta-analyses: for their ability to synthesize high-level evidence.
- Randomized controlled trials (RCTs): considered the gold standard for evaluating intervention effectiveness.
- Cohort studies: useful for examining the long-term impact of SC in real-world settings where RCTs are not always feasible.

### 2.6. Search Strategy and Descriptors

The search strategy used controlled vocabulary from the Medical Subject Headings (MeSH) and Health Science Descriptors (DeCS), combining terms with Boolean operators (AND, OR, NOT) to increase both sensitivity and specificity.

### 2.7. Data Analysis and Quality Assessment

- The methodological quality of the included studies was evaluated using specific tools depending on study design:
- JADAD Scale: for randomized controlled trials, assessing randomization, blinding, and loss to follow-up (5 items).
- AMSTAR 2 (A Measurement Tool to Assess Systematic Reviews): for systematic reviews and meta-analyses (16 items).
- Newcastle-Ottawa Scale (NOS): for cohort studies, evaluating patient selection, comparability, and outcomes (9 items).

In parallel, the risk of bias was evaluated using validated tools:

- ROBINS-I: for non-randomized studies of interventions (Appendix Table A4).
- ROBIS: for systematic reviews (Appendix Table A5).
- RoB 2: for randomized trials (Appendix Table A6).

### 2.8. Study Selection and Data Synthesis

The study selection process followed the PRISMA 2020 flowchart and was conducted in three stages (Figure S1):

1. Eligibility definition and application of inclusion/exclusion criteria
2. Identification and screening: removal of duplicates and preliminary screening of titles and abstracts
3. Full-text evaluation: assessing alignment with objectives and verifying methodological quality

The final synthesis integrated the most relevant findings concerning SC effectiveness, functional prognosis, and the role of nursing in neurorehabilitation.

## 3. Results

Following the implementation of the search strategy, a total of 13 articles meeting the established inclusion criteria were selected for this review, comprising an overall sample of 80,555 patients (Figure S1).

The results of the included studies are presented according to the objectives outlined in the methodology.

### 3.1. Analysis of the Impact of Stroke Code (SC) Care Times on Functional Recovery, and Identification of Factors Affecting Time Optimization

An initial approach to this question is provided by the study of Soto-Cámara et al. [18], which analyzed the determinants of prehospital time (PHT) in stroke care, grouping them into five categories: sociodemographic, clinical, contextual, behavioral, and cognitive. Early medical attention was favored by greater stroke severity (OR: 1.04–9.17), due to the increased perception of urgency; the presence of cardiovascular diseases (OR: 1.08–3.84); or the correct identification of symptoms as characteristic of stroke (OR: 1.13–4.57). Conversely, longer delays were associated with diabetes mellitus (OR: 1.25–4.35), due to the tendency to misinterpret symptoms as hypoglycemia; geographical setting (OR: 1.14–5.00); or referral from non-specialized centers for reperfusion therapies (OR: 1.43–16.79).

Along similar lines, the study by Darehead et al. [19], with a sample of 14,132 patients, quantified the clinical impact of door-to-needle time (DNT). Findings indicated that each minute of delay was associated with a 0.6% reduction in 90-day survival (OR 0.994), a 0.3% increase in the risk of intracerebral hemorrhage within 36 h (OR 1.003), as well as a 0.3% worsening in activities of daily living (ADL) (OR 1.003) and a 0.4% decline in mobility at 3 months (OR 1.004).

In support, Man et al. [20] evaluated a cohort of 61,426 patients treated with rt-PA (recombinant tissue plasminogen activator) within 4.5 h and assessed outcomes at one year. Patients were stratified by DNT at 45 and 60 min. Among those with DNT exceeding 45 min, 79.2% had significantly higher mortality (35.0% vs. 30.8%) and higher readmission rates (40.8% vs. 38.4%) compared to those treated sooner. Similar findings were observed in the 60-min group. Each 15-min increase in DNT was associated with a 4% increase in mortality and a 2% rise in all-cause readmission (adjusted HR: 1.04 and 1.02, respectively), a significant relationship only when treatment was given within the first 90 min, with no notable benefit for times under 30 min.

**Figure 1:**
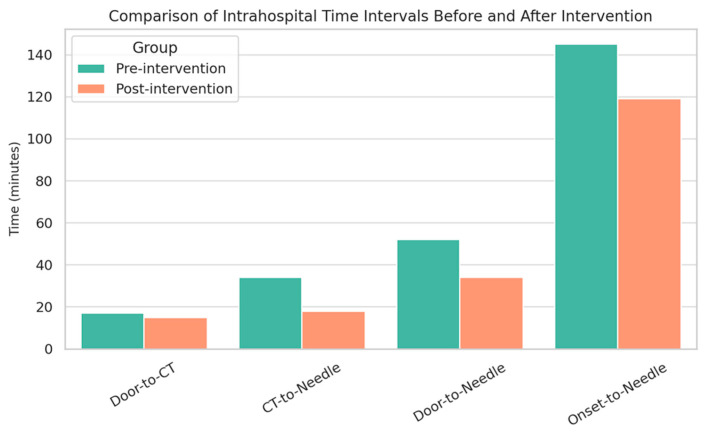
Comparison of In-hospital Stroke Care Times Before and After Intervention (Pre-intervention (minutes) Post-intervention (minutes)).

Regarding strategies to optimize treatment times, the meta-analysis by Mohedano et al. [21] demonstrated that a new in-hospital protocol significantly reduced DNT in patients who have suffered an acute stroke treated with IVT, achieving times below 30 min. A total of 239 patients were included in the pre-intervention group and 222 in the post-intervention group. After implementing the new protocol, all in-hospital time metrics were significantly reduced (*p* < 0.001), except for onset-to-door time (Figure 1). The reduction was most pronounced in patients with prehospital SC activation, with a median of 22 min.

Consistently, Wang et al. [22] investigated the impact of implementing an emergency nursing pathway in acute stroke patients. A final sample of 104 patients was randomly assigned to an intervention group (*n* = 53) that followed the emergency nursing protocol, and a control group (*n* = 51) that received routine emergency care. The nursing pathway included three main components. The intervention group had significantly shorter onset-to-door (ODT) and DNT times (*p* < 0.05). After two weeks, the NIHSS score showed a greater reduction in the intervention group than in the control group (*p* < 0.05).

### 3.2. To Evaluate the Effectiveness of In-Hospital Interventions Implemented Under the SC

A comparative analysis was conducted to evaluate the efficacy of key ischemic stroke reperfusion strategies (IVT and MT) on functional outcomes. In this context, Zhang et al. [23] conducted a clinical trial to assess the effectiveness of rt-PA therapy compared with a combination of aspirin and clopidogrel. Patients were randomly assigned to two groups: one received only oral antiplatelet agents (*n* = 38), and the other received additional rt-PA (*n* = 38). The rt-PA group exhibited greater neurological improvement (*p* < 0.05). Moreover, they showed significantly lower levels of inflammatory markers (Lp-PLA2, HCY, and hsCRP), and better coagulation profiles (lower vWF and factor VIII levels). No significant differences in adverse event incidence were found (*p* > 0.05), suggesting that the treatment is safe. Regarding prognosis, 63.2% of rt-PA patients experienced better clinical outcomes versus 31.6% in the conventional group (*p* < 0.05).

Similarly, Muir et al. (ATTEST-2) [24] compared Tenecteplase (0.25 mg/kg) and alteplase (0.9 mg/kg) in treating acute ischemic stroke within 4.5 h of symptom onset, aiming to determine Tenecteplase efficacy in a thrombolysis-eligible population. A total of 1,777 patients were included (885 Tenecteplase, 892 alteplase). The primary outcome, 90-day modified Rankin Scale (mRS), showed an adjusted odds ratio of 1.07, confirming the non-inferiority of Tenecteplase (*p* < 0.0001), with no superiority evidence (*p* = 0.43). For secondary outcomes, the proportion of patients with excellent recovery (mRS 0–1) was 44% (Tenecteplase) vs. 42% (alteplase) (OR 1.05), and functional independence (mRS 0–2) was achieved in 68% vs. 65% (OR 1.15), both without significant differences. Mortality rates were similar (8% in both groups, HR 0.96).

Fischer et al. (SWIFT DIRECT) [25] compared thrombectomy alone versus thrombectomy plus intravenous alteplase in 408 randomized patients (201 thrombectomy-only, 207 IV alteplase + thrombectomy). At 90 days, mRS scores of 0–2 were achieved in 57% of the thrombectomy-only group and 65% in the combination group (ARD −7.3%). Mortality was higher in the thrombectomy-only group (11% vs. 9%). In terms of mobility, the thrombectomy-only group had a higher proportion of patients with limitations (47% vs. 37%), as well as higher rates of issues in self-care (32% vs. 29%) and pain/discomfort (54% vs. 44%). There were no significant differences in adverse effects, including symptomatic intracranial hemorrhage (2% vs. 3%). However, successful reperfusion was lower in the thrombectomy-only group (91% vs. 96%, *p* = 0.047).

Sarraj et al. (SELECT2) [26] analyzed the relationship between imaging-based estimates of irreversible brain injury (core) and at-risk regions (mismatch), as well as their impact on clinical outcomes and endovascular treatment in ischemic stroke patients. In a sample of 336 patients without pre-stroke disability, greater central perfusion volume on CT was associated with worse functional outcomes in those undergoing thrombectomy (aGenOR: 0.92 per 10 mL increase).

Increased age (aRR: 0.97 per year) and time from CT-based occlusion diagnosis to reperfusion (aRR: 0.97 per 10-min delay) were significantly associated with a reduced likelihood of independent ambulation.

### 3.3. Contribution of Nursing in the Recovery Process Through Involvement in Inpatient Neurorehabilitation Programs

**Figure 2:**
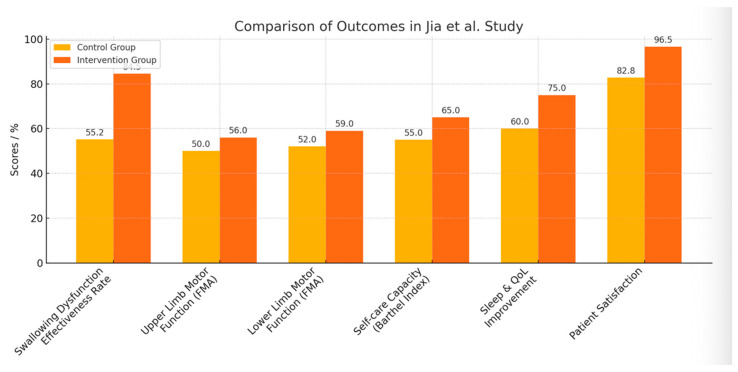
Impact of Nurse-Led Early Rehabilitation on Stroke Patient Recovery Outcomes. The figure date is from Jia et al. [27].

Within the framework of inpatient rehabilitation, several studies have analyzed the influence of early intervention and the active role of nursing staff. In this regard, the clinical trial conducted by Jia et al. [27] on the effects of early nurse-led rehabilitation on motor function, swallowing, and quality of life in stroke patients showed a positive impact on recovery in the intervention group (IG; *n* = 58) compared to the control group (CG; *n* = 58). The IG demonstrated a higher effectiveness rate in the management of swallowing dysfunction, with a significant effect of 84.49% versus 55.17% in the CG; motor function in both upper and lower limbs improved significantly according to the Fugl-Meyer Assessment (FMA), with a mean difference of approximately 6 points in the upper limb and 7 in the lower limb compared to the control group. Moreover, the IG achieved greater recovery in self-care capacity according to the Barthel Index (BI). Similarly, both sleep quality and overall quality of life improved more markedly in the IG, as did patient satisfaction with care (IG: 96.55%; CG: 82.76%) (Figure 2).

In line with these findings, the study by Wang et al. [28] evaluated the feasibility of a nurse-led motor function rehabilitation program developed according to Orem’s self-care theory. The intervention consisted of 7 consecutive days of training, twice daily for 30 min. The experimental group (*n* = 43) received training in activities of daily living and early mobilization, while the control group (*n* = 45) received standard care. The experimental group exhibited more pronounced improvements than the control group. In the BI, the median increased from 36 to 62 points in the experimental group, compared to an increase from 39 to 54 in the control group. On the Motor Assessment Scale (MAS), the experimental group improved from 16 to 30 points, while the control group progressed from 16 to 24. Both differences were statistically significant (*p* = 0.002 and *p* = 0.001, respectively). Regarding the NIHSS scale, both groups showed a reduction in score (indicating an improvement in stroke severity), but no significant differences were observed between them (*p* = 0.673).

From a broader perspective, Tanlaka et al. [29] analyzed the nursing role in stroke rehabilitation units, highlighting three key areas of involvement: support in activities of daily living (hygiene, nutrition, mobility, and skin care); administration and monitoring of therapeutic interventions (medication, vital signs, and wound care); and education and emotional support for patients and families, aiming to promote self-care and independence. Nevertheless, the study also identified barriers such as lack of time, staff shortages, and insufficient training in stroke-specific care.

Finally, the meta-analysis by Gu et al. [30] evaluated the effects of nursing involvement in early rehabilitation on neurological function and quality of life in patients with hemiplegia. The sample included a total of 1,631 subjects, divided into a control group (*n* = 806), which received routine nursing care (routine medication and basic exercises), and an observation group (*n* = 825) that underwent early rehabilitation with active nursing involvement (health education, daily living skills training, limb exercises, psychological support, dietary guidance, cognitive intervention, and family support). The findings indicated that early rehabilitation intervention significantly improved various indicators: NIHSS scale (SMD = -1.623), FMA scale (SMD = 2.688), BI (SMD = 1.617), and care satisfaction (RR = 1.191), among others, compared to the control group receiving standard care.

## 4. Discussion

The analysis of the selected literature demonstrates that Stroke Code interventions have a positive impact on the functional prognosis of patients who have suffered an acute ischemic stroke. Two key aspects were analyzed: on the one hand, the efficiency and impact on time management [18,19,20,21,22], and on the other, the outcomes of reperfusion therapies [23,24,25,26]. In addition, the reviewed studies highlight the fundamental role of nursing professionals in hospital-based rehabilitation [27,28,29,30].

Prehospital management is crucial for optimizing care, as it directly influences the timely application of reperfusion treatments and, consequently, the functional recovery of the patient. The identification of influencing factors in first-contact response times by Soto-Cámara et al. [18] underscores the need for measures aimed at reducing delays, improving care efficiency, and promoting public education, given that bystanders are often the first to witness symptom onset.

Multiple studies have confirmed the relationship between DNT (door-to-needle time) and patient prognosis. Darehed et al. [19] and Man et al. [20] concur that even marginal reductions in DNT significantly impact patient mortality and functional outcomes. Both authors emphasize the importance of minimizing all delays, as early restoration of cerebral blood flow is essential to limit neuronal damage. However, despite the benefits of shorter intervention times, there are boundaries and areas that warrant further investigation. In Man’s study [20], the lack of significant improvement in outcomes with DNTs under 30 min suggests saturation points where further reductions yield minimal additional benefit. Darehed [19] also notes that factors such as age, NIHSS score, and ODT (onset-to-door time) influence treatment times, indicating that not all delays are equal and some may lie beyond the immediate control of healthcare professionals.

The treatment of acute ischemic stroke is an evolving field. The analyzed RCTs reflect the effects of reperfusion strategies on functional outcomes. Zhang’s trial [23] confirms the superiority of intravenous thrombolysis with rt-PA over antiplatelet therapy, demonstrating improved neurological recovery and functional prognosis. These benefits may be attributed to modulation of inflammatory biomarkers and restoration of coagulation balance. If rt-PA reduces inflammation, it could contribute to limiting the extent of cerebral damage, while rebalancing coagulation may accelerate recovery and reduce adverse events.

The ATTEST-2 trial [24] complements this perspective by comparing alteplase with Tenecteplase. While alteplase remains an effective therapeutic option, Tenecteplase emerges as a viable and more logistically convenient alternative. Its single intravenous bolus administration streamlines the thrombolytic process compared to alteplase, which requires an initial bolus followed by a continuous one-hour infusion. Clinical trials such as AcT [31] have supported the therapeutic equivalence of both agents, while others, such as EXTEND-IA TNK [32], suggest a possible superiority of Tenecteplase in terms of functional outcomes.

Regarding thrombectomy, the SWIFT-DIRECT study [25] concludes that combining mechanical thrombectomy with intravenous alteplase yields better reperfusion and clinical recovery outcomes than thrombectomy alone. These results align with previous trials such as MR CLEAN-NO IV and SKIP [33,34] but contrast with DIRECT MT and DEVT [35,36], which suggest that direct thrombectomy is not inferior to combination therapy with rt-PA, highlighting the need for further studies to clarify these discrepancies.

The SELECT-2 study [26] evaluated the benefit of endovascular thrombectomy in patients with large ischemic cores. While thrombectomy showed benefits across a broad range of ischemic volumes, outcomes were poorer in patients with core volumes greater than 100 mL. Approximately 80% of these patients experienced moderate to severe disability (mRS score ≥4) at 90-day follow-up. However, the fact that one in five patients regained independent ambulation emphasizes that, despite limited benefits in certain subgroups, functional improvement remains possible. This potential increases in younger patients or those receiving early treatment. These findings reinforce the importance of early diagnosis and rapid intervention to maximize recovery opportunities.

Beyond the treatment of choice, functional recovery greatly depends on follow-up and rehabilitation. Two RCTs [27,28] and one meta-analysis [30] on different rehabilitation areas related to post-stroke sequelae were included. These studies highlight the benefits of nursing intervention in neurological rehabilitation, showing improvements in functionality and quality of life. They contribute a holistic approach and tailored rehabilitative strategies adapted to individual needs, supporting more effective recovery. The importance of early rehabilitation (within the first 24 h post-stabilization) [30] is also emphasized due to its impact on neuroplasticity and neuronal recovery, although the optimal timing remains a challenge due to the need to balance risks and benefits.

In contrast, Tanlaka et al. [29], while acknowledging the relevance of nursing care, point to the limited visibility of the nursing role in stroke rehabilitation. This is attributed to the lack of standardized competencies and interdisciplinary recognition. Previous studies, such as that by Ehrlich et al. [37], support this view, indicating that ambiguity in nursing roles limits their potential impact on hospital care [38,39,40,41].

This review has several limitations that should be acknowledged. Firstly, the heterogeneity of the included studies—both in design and measured variables—complicates the comparison of certain results. Additionally, there is a notable lack of research on the nursing role in the hospital rehabilitation context, limiting an in-depth analysis of this area.

Nonetheless, this review enables a comprehensive understanding of how each phase of the care process influences the patient’s functional prognosis and emphasizes the importance of continuity of care following the acute phase of stroke treatment.

## 5. Conclusions

This systematic review highlights the substantial impact of Stroke Code interventions on the functional outcomes of patients who have suffered an acute ischemic stroke. Timely and efficient management—particularly in regard to prehospital care and door-to-needle times—plays a decisive role in improving survival and long-term neurological function. The evidence supports that even marginal reductions in treatment delays lead to significantly better prognoses, underscoring the importance of continuous optimization of emergency workflows and public awareness.

Reperfusion therapies, including intravenous thrombolysis and mechanical thrombectomy, demonstrate clear benefits, although the choice of strategy and its timing must be tailored to individual patient profiles. The emergence of Tenecteplase as a viable alternative to alteplase and the expanding indications for thrombectomy in patients with large ischemic cores open new avenues for personalized stroke care. However, variability in outcomes among recent clinical trials indicates the need for further high-quality research to define best practices across different clinical scenarios.

Importantly, the role of nursing professionals extends beyond the acute phase, contributing significantly to functional recovery through individualized and early rehabilitation interventions. Despite the evidence supporting the effectiveness of nursing-led strategies, the lack of standardized competencies and visibility within interdisciplinary teams continues to limit their impact.

In conclusion, an integrated, time-sensitive, and patient-centered approach—supported by well-coordinated emergency systems and empowered nursing roles—is essential to improving functional prognosis after an ischemic stroke. Addressing current gaps in rehabilitation research and reinforcing continuity of care are key to enhancing stroke recovery outcomes on a system-wide level.

## Data Availability

Not applicable. No new data were created.

## Supplementary Materials

The following supporting information can be downloaded at: https://www.mdpi.com/article/10.3390/neurolint17110175/s1, Figure S1: PRISMA 2020 flow diagram.

## Appendix

**Table A1:** List of MeSH and DeCS terminology used in the searches and subsequently combined with Boolean operators.

Category	MeSH Terms	DeCS Terms
Clinical condition	Acute Stroke	Acute stroke
	Stroke	Ischemic stroke
Factors and prognosis	Prognosis	Treatment Efficacy
	Time Factors	Treatment Outcome
Interventions and care	Nursing Care	Clinical nursing research
	Rehabilitation	Door-to-Treatment Time
		Thrombolysis
		Thrombectomy
		Quality improvement
Healthcare personnel		Nurse
		Nurs *

*: Use of wildcards to capture terms such as nursing, nurses, etc.

**Table A2:** Search strategies used in the databases employed. Terms and Boolean operators were adapted to the specifics of each platform.

Database	Search Strategy
**PubMed**	(((stroke[MeSH Terms]) AND (time factor [MeSH Terms])) AND (prognosis[MeSH Terms])) AND (after treatment [MeSH Terms])
**Scopus**	(TITLE-ABS-KEY(“stroke”) AND TITLE-ABS-KEY(“functional AND prognosis”) AND TITLE-ABS-KEY(“nurs*”) AND TITLE-ABS-KEY(“rehabilitation”))
**Cochrane**	(“Stroke”[MeSH Terms] OR “Acute Stroke”[Title/Abstract]) AND (“Functional Status”[MeSH Terms] AND (“Prognosis”[MeSH Terms])
**Dialnet**	ictus isquémico, pronóstico funcional, código ictus, neurorrehabilitación y enfermería
**Web of** **Science**	TS = (“stroke” AND “code”) AND TS = (“functional prognosis”) AND TS = (“nursing”) AND TS = (“rehabilitation”)

**Table A3:** Studies included in the review along with the source database, article design and assessment of methodological quality.

Title	Database	Type of Article	Level of Methodological Quality
“Analysis of factors related to prehospital time in stroke care” [18]	Dialnet	Systematic review	AMSTAR: 14
“In-Hospital Delays in Stroke Thrombolysis: Every Minute Counts” [19]	Scopus	Retrospective cohort study	NOS: 9
“Association Between Thrombolytic Door-to-Needle Time and 1-Year Mortality and Readmission in Patients With Acute Ischemic Stroke” [20]	PubMed	Retrospective cohort study	NOS: 9
“A new in-hospital protocol reduces door-to-needle time in acute stroke treated with intravenous thrombolysis to less than 30 min.” [21]	Dialnet	Retrospective cohort study	NOS: 8
“Effect and prognosis of emergency nursing path in patients with acute stroke” [22]	Cochrane	Retrospective cohort study	NOS: 9
“Recombinant tissue-type plasminogen activator (rt-PA) effectively restores neurological function and improves prognosis in acute ischemic stroke” [23]	PubMed	Randomized clinical trial	JADAD: 3
“Tenecteplase versus alteplase for acute stroke within 4·5 h of onset (ATTEST-2): A randomised, parallel group, open-label trial” [24]	PubMed	Randomized clinical trial	JADAD: 5
“Thrombectomy alone versus intravenous alteplase plus thrombectomy in patients with stroke: An open-label, blinded-outcome, randomised non-inferiority trial” [25]	PubMed	Randomized clinical trial	JADAD: 5
“Endovascular Thrombectomy for Large Ischemic Stroke Across Ischemic Injury and Penumbra Profiles” [26]	PubMed	Randomized clinical trial	JADAD: 3
“Influences of Early Rehabilitation Nursing on Motor Function, Swallowing Function as Well as Quality of Life in Stroke Patients” [27]	Cochrane	Randomized clinical trial	JADAD: 3
“Nurse-Led Motor Function Rehabilitation Program for Acute Ischemic Stroke: A Randomized Pilot Study” [28]	WoS	Randomized clinical trial	JADAD: 3
“The Role and Contributions of Nurses in Stroke Rehabilitation Units: An Integrative Review” [29]	PubMed	Systematic review	AMSTAR: 14
“Effect of early rehabilitation nursing on neurological function and quality of life of patients with hemiplegia after stroke: A meta-analysis” [30]	Wos	Meta-analysis	AMSTAR: 12

**Table A4:** Assessment of risk of bias using the ROBINS-I scale (1 (Low risk of bias); 2 (Moderate risk of bias); 3 (High risk of bias)).

	Darehead et al. (2020) [19]	Man et al. (2020) [20]	Mohedano et al. (2021) [21]	Wang et al. (2021) [22]	Gu et al. (2023) [30]
D1: Bias due to confounding factors	1	1	2	2	1
D2: Bias due to participant selection	1	1	1	1	1
D3: Bias in the classification of interventions	1	1	1	1	2
D4: Bias due to deviations from the planned interventions	1	1	1	1	2
D5: Bias due to missing data	1	1	1	2	1
D6: Bias in the measurement of results	1	1	1	3	1
D7: Bias in the selection of the reported outcome	1	1	1	1	1
Overall assessment	1	1	1	2	2

**Table A5:** Assessment of risk of bias using the ROBIS scale (1 (Low risk of bias); 2 (Moderate risk of bias); 3 (High risk of bias)).

	Soto-Cámara et al. (2020) [18]	Tanlaka et al. (2023) [29]
D1: Relevance of eligibility criteria	1	1
D2: Study identification and selection	1	1
D3: Data collection and assessment of risk of bias in individual studies	1	2
D4: Synthesis and findings	1	1
D5: Interpretation of findings	1	1
D6: Funding and conflicts of interest	1	1
Overall assessment	1	1

**Table A6:** Assessment of risk of bias using the RoB 2 scale (1 (Low risk of bias); 2 (Moderate risk of bias); 3 (High risk of bias)).

	Zhang et al. (2023) [23]	Muir et al. (2024) [24]	Fischer et al. (2022) [25]	Sarraj et al. (2024) [26]	Jia et al. (2024) [27]	Wang et al. (2022) [28]
D1: Bias in the randomization process	2	1	1	1	2	1
D2: Bias due to deviations from the intended interventions	3	2	2	2	1	2
D3: Bias due to missing outcome data	1	1	1	1	1	2
D4: Bias in outcome measurement	2	1	1	1	2	2
D5: Bias in the selection of the reported outcome	1	1	1	1	1	1
Overall assessment	2	1	1	1	2	2

**Table A7:** Summary table of the studies included in the review.

Author/Year/ Country	Sample	Objective	Key Results	Conclusions
**Soto-Cámara** **et al. (2021)–** **España** [18]	**—**	Identify the factors that influence prehospital time (PHT) in patients with stroke.	Immediate notification to the emergency medical system (EMS) was the most determining factor in reducing TPH, while referral from another health center was associated with an increase in said time.	HSCT is influenced by patient and environmental factors, including demographic, clinical, behavioral, and cognitive aspects. Implementing educational strategies, encouraging early symptom recognition, and promoting immediate EMS notification can contribute to reducing these times.
**Darehed et** **al. (2020)–** **Suecia** [19]	**1.4132**	To study the impact of time factors on thrombolysis and how it influences the improvement of the quality of stroke care.	Each minute of delay in door-to-needle time decreases the chances of survival by 0.6%, increases the chances of intracerebral hemorrhage and worse functional results in ADL by 0.3%, and produces a 0.4% worsening in living conditions and mobility.	Early intervention and optimization of treatment time are essential to improve the quality of care and outcomes in stroke.
**Man et al.** **(2020)–** **EE.UU.** [20]	**6.1426**	To examine whether shorter door-to-needle times with rt-PA treatment are associated with better long-term outcomes.	Shorter TPAs were associated with better long-term outcomes: Lower mortality and all-cause readmission rates at one year.	The study’s findings reinforce efforts to shorten the time to thrombolytic therapy.
**Mohedano** **et al. (2021)–** **España** [21]	**461**	Implement measures to reduce intervention time in patients with acute ischemic stroke in in-hospital settings.	The implementation of the new protocol achieved a median reduction in the time from symptom onset to IVD by 26 min.	The goal of treating the patient within the first 60 min is outdated, so more ambitious goals should be sought to reduce door-to-needle times. Speedy treatment administration is crucial to improving functional outcomes.
**Wang et al.** **(2021) -** **China** [22]	**104**	To evaluate the effect and prognosis of nursing in the emergency setting of stroke.	In the observation group, the time from admission to diagnosis and from diagnosis to specialized treatment were significantly shorter. Furthermore, the scales used to assess cognitive and neurological outcomes showed more notable improvements in this group, which also had a lower rate of disability.	The incorporation of emergency nursing between admission and specialized treatment, promoting neurological and cognitive recovery.
**Zhang et al.** **(2023)–** **China** [23]	**76**	To evaluate the efficacy of intravenous thrombolysis with rt-PA in ischemic stroke in addition to oral drug treatment (clopidogrel and aspirin).	Patients treated with rt-PA showed improved neurological function at 24 h (lower NIHSS scores) and reduced mortality, compared with conventional drug therapy without rt-PA.	The addition of thrombolysis with rt-PA to oral treatment optimized the clinical outcome and prognosis of patients, promoting neurological recovery without increasing the risk of adverse effects.
**Muir et al.** **(2024)–** **Reino Unido** [24]	**1777**	To examine the potential advantages of Tenecteplase over alteplase within 4.5 h of stroke onset.	Superiority of Tenecteplase over alteplase was not demonstrated for primary and secondary outcomes, however mRS scores at day 90 favored Tenecteplase.	The ATTEST-2 study corroborates the non-inferiority of Tenecteplase compared to alteplase in acute ischemic stroke. Considering the greater ease of administration of Tenecteplase (single IV bolus), it may help reduce door-to-needle times.
**Fischer et al.** **(2022)–** **Multinacional** **(Europa,** **EE.UU. y** **Canadá)** [25]	**408**	To determine the efficacy of endovascular treatment with thrombectomy compared to IV alteplase plus thrombectomy in ischemic stroke, evaluating functional independence at 3 months.	The group receiving both treatments (thrombectomy and fibrinolysis with alteplase) had better outcomes measured by mRS, quality of life, and successful reperfusion. The occurrence of serious adverse events did not differ significantly between the two groups.	Non-inferiority of thrombectomy alone versus thrombectomy and IV alteplase combined was not demonstrated. Based on these results, omitting IV alteplase therapy is not recommended in eligible patients.
**Sarraj et al.** **(2024)–** **Multinacional** **(EE.UU. y** **otros países)** [26]	**336**	To evaluate the relationship between imaging estimates of the irreversibly injured brain (core) and regions at risk (mismatch), as well as the association of this mismatch with clinical outcomes and the effect of endovascular thrombectomy in acute ischemic stroke.	Patients who received thrombectomy had better clinical outcomes, according to the mRS, compared to those who received only conventional medical care.	Thrombectomy improves clinical outcomes across a broad spectrum of infarct volumes; however, clinical outcomes worsen as the extent of the ischemic lesion increases.
**Jia et al. (2024)–** **China** [27]	**116**	To investigate the effectiveness of early rehabilitation with nursing involvement in motor function, swallowing, and quality of life of patients.	The research group showed a significantly higher overall recovery rate in swallowing dysfunction, recovery of limb function, self-care capacity, quality of sleep and life, as well as overall satisfaction with treatment.	The effectiveness of nursing in early rehabilitation contributes significantly to the improvement of swallowing and limb dysfunction. Therefore, it should be considered in clinical practice.
**Wang et al. (2022)–China** [28]	**88**	To evaluate the feasibility of a nurse-led motor function rehabilitation program based on Orem’s theory.	The experimental group showed significant improvements in the Barthel Index and the Motor Assessment Scale compared to the control group.	A nurse-led rehabilitation program based on Orem’s theory is effective in improving motor function in post-stroke patients.
**Tanlaka et al. (2023)–Canadá** [29]	**—**	To analyze the role and contributions of nurses in stroke rehabilitation units.	Nurses play key roles in daily activities, therapeutic monitoring, and emotional support. However, they face challenges such as lack of time and personnel.	It is essential to recognize and support the multifaceted role of nurses in the rehabilitation of stroke patients.
**Gu et al. (2023)–China** [30]	**1631**	To evaluate the effects of early rehabilitation nursing on neurological function and quality of life in patients with post-stroke hemiplegia.	Early rehabilitation intervention significantly improved neurological function, independence in daily activities, and satisfaction with care.	Early nursing-led rehabilitation is crucial to improve functional outcomes and quality of life in patients with post-stroke hemiplegia.
