# Correction: Astasio-Picado et al. Impact of Stroke Code Activation on Functional Outcomes and the Role of Nursing in Neurorehabilitation: A Systematic Review. *Neurol. Int.* 2025, *17*, 175

**DOI:** 10.3390/neurolint18020035

**Published:** 2026-02-12

**Authors:** Álvaro Astasio-Picado, Jesus Jurado-Palomo, Clara Fátima Rodriguez-Urbaneja

**Affiliations:** 1 Physiotherapy, Nursing and Physiology Department, Faculty of Health Sciences, University of Castilla-La Mancha, 45600 Toledo, Spain; jesus.juradopalomo@uclm.es (J.J.-P.); clarafatima.urbaneja@gmail.com (C.F.R.-U.); 2 Department of Nursing, University Center of Plasencia, University of Extremadura, 10600 Cáceres, Spain

**Missing Supplementary File**

In the original publication [1], a supplementary file, “PRISMA 2020 Flow Diagram”, was not published. This supplementary file has been added to the online version. The detailed description is as follows: The following supporting information can be downloaded at https://www.mdpi.com/article/10.3390/neurolint17110175/s1, Figure S1: PRISMA 2020 flow diagram.

**Text Correction**

In the original publication, the author forgot to cite Figure S1 in Sections 2.8 and 3. The correct description is as follows:

“The study selection process followed the PRISMA 2020 flowchart and was conducted in three stages (Figure S1):”

“Following the implementation of the search strategy, a total of 13 articles meeting the established inclusion criteria were selected for this review, comprising an overall sample of 80,555 patients (Figure S1).”

The authors state that the scientific conclusions are unaffected. This correction was approved by the Academic Editor. The original publication has also been updated.
