# Supplementary figures and images for: Impact of Stroke Code Activation on Functional Outcomes and the Role of Nursing in Neurorehabilitation: A Systematic Review
Source: Neurol Int. 2025 Oct 29;17(11):175. doi: 10.3390/neurolint17110175 (PMC12655110; doi:10.3390/neurolint17110175)

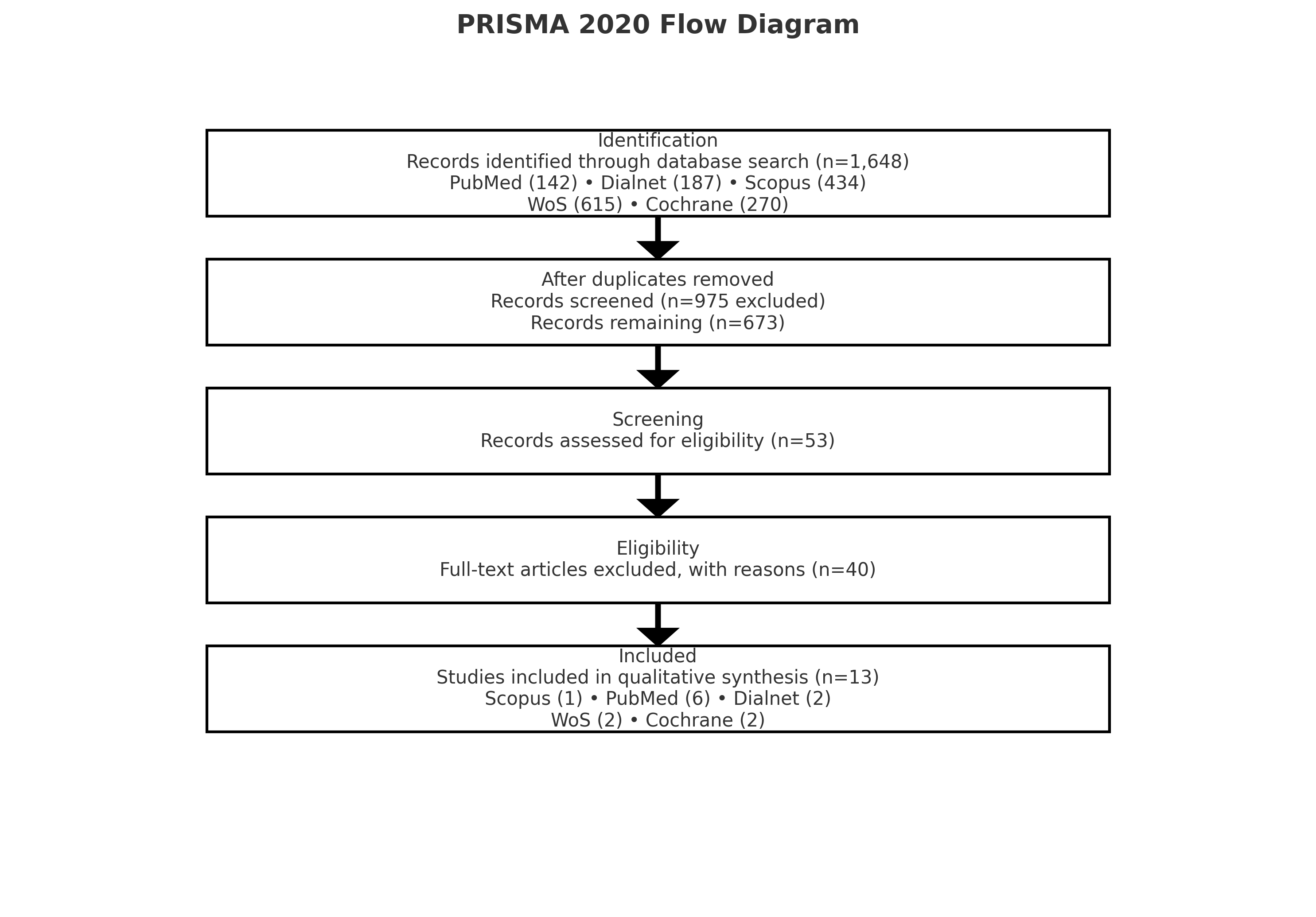

Supplement: Supplementary file 1 [file neurolint-17-00175-s001.zip › neurolint-3914293-supplementary.png]
